# Antioxidants and molecular damage in Nile Tilapia (*Oreochromis niloticus*) after exposure to microplastics

**DOI:** 10.1007/s11356-020-07898-y

**Published:** 2020-02-11

**Authors:** Mohamed Hamed, Hamdy A. M. Soliman, Alaa G. M. Osman, Alaa El-Din H. Sayed

**Affiliations:** 1 Department of Zoology, Faculty of Science, Al-AzharUniversity (Assiut Branch), Assiut, 71524 Egypt; 2 Department of Zoology, Faculty of Science, Sohag University, Sohag, 8562 Egypt; 3 Department of Zoology, Faculty of Science, Assiut University, Assiut, 71516 Egypt

**Keywords:** Microplastics, Nile tilapia, Malondialdehyde, DNA damage, SDS-PAGE

## Abstract

Recently, research on the biological effects of microplastics (MPs) has grown exponentially. However, effects of MPs on freshwater fishes and the mechanisms of the biological effects of MPs were limited. So, the purpose of the current study was to clarify the effects of microplastics on oxidative stress response, DNA fragmentation, and proteinogram of the early juvenile stage of Nile Tilapia (*Oreochromis niloticus*). The fishes were assigned into four groups: one control, three MPs-exposed groups as 1 mg/L of MPs, 10 mg/L of MPs, and 100 mg/L of MPs respectively for 15 days and 15 days of recovery. The activities of superoxide dismutase, catalase, total peroxides, and oxidative stress index (OSI), as well as lipid peroxidation and DNA fragmentation, increased in groups exposed to MPs compared to the control group in a dose-dependent manner. In contrast, the activity of total antioxidant capacity decreased in groups exposed to MPs compared to the control group in a dose-dependent manner. The electrophoretic pattern of muscle proteins revealed alteration in the proteinogram in the MPs-exposed groups compared to control. After the recovery period, the activities of superoxide dismutase, catalase, total peroxides, total antioxidant capacity, lipid peroxidation, DNA fragmentation, and the electrophoretic pattern of muscle proteins returned to normal levels in 1 mg/L of MPs-exposed group. Combined with our previous work, these results suggest that MPs cause the overproduction of reactive oxygen species (ROS) and alters the antioxidants parameters, resulting in oxidative stress and DNA damage. The present study fosters a better understanding of the toxic effects of MPs on Tilapia as a freshwater model.

**Figure:**
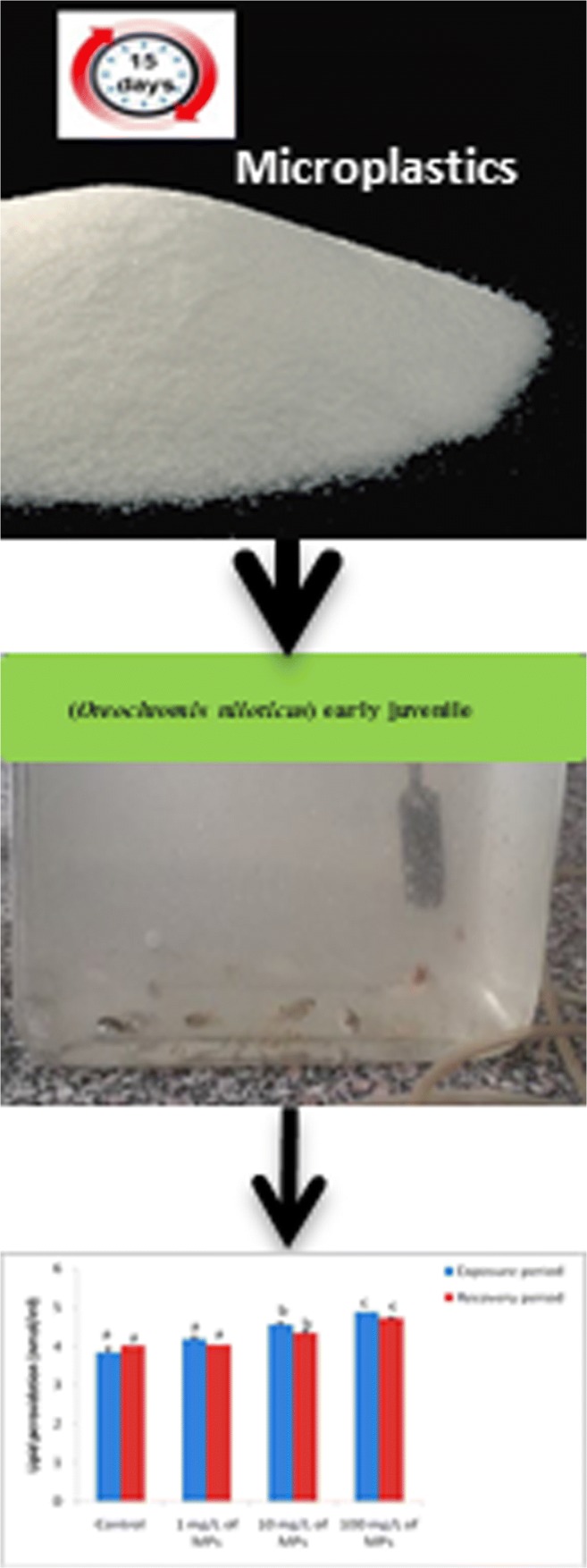
Graphical Abstract

Graphical Abstract

## Introduction

Recently, there is increasing sensibility of the harmful effects of microplastics due to their extensive use in commercial, industrial, and medicinal applications (Lusher et al. 2017). It is known that exposure of fish to different pollutants in the water ecosystem can prompt the surfeit of reactive oxygen species (ROS) which causes harmful effects to macromolecules of cells (Sureda et al. 2006) because microplastics can be digested by fish and causes some effects (de Sá et al. 2018; Jovanovic 2017). The fish have an intricate antioxidant system which comprises superoxide dismutase (SOD), catalase (CAT), glutathione S-transferase (GST), and glutathione peroxidase (GPx), which encounter the oxidative damage of reactive oxygen species (ROS) (Sayed and Soliman 2018; Soliman et al. 2019). Oxidative stress index (OSI) is a comparative indicator illustrating the interaction among the free-radical inducing agents and their antioxidants system (Fazio et al. 2015; Sayed and Abul Khalil 2016). Malondialdehyde (MDA) is regarded as a bioindicator of the lipid peroxidation (LPO) (Karami et al. 2016). The induction of antioxidant enzymes and the elevation of the lipid peroxidation in fish exposed to different pollutants can be regarded as bioindicators of oxidative stress (Karami et al. 2016). Microplastics may induce oxidative stress by different mechanisms as stimulating some intracellular signal transduction pathways as in *Brachionus koreanus* (Jeong et al. 2016) and zebrafish (*Danio rerio*) (Lu et al. 2016), activation of catalase in sheepshead minnows (*Cyprinodon variegatus*) (Choi et al. 2018), elevation of brain lipid peroxidation (LPO) levels in juveniles of the European bass (*Dicentrarchus labrax*) (Barboza et al. 2018), suppression of antioxidant enzymes in juvenile of the Chinese mitten crab (*Eriocheir sinensis*) (Yu et al. 2018), decline the catalase levels and the glutathione content in zebrafish larvae (Wan et al. 2019), induction of superoxide dismutase (SOD) levels in liver of red tilapia (*Oreochromis niloticus*) (Ding et al. 2018; Zhang et al. 2018), and the upregulation of superoxide dismutase (SOD) and catalase (CAT) levels in the gut of the zebrafish (Qiao et al. 2019).

DNA fragmentation assay is regarded as a valuable method to evaluate the genotoxicity of any pollutant in the environment (Frenzilli et al. 2009; Nacci et al. 1996). Microplastics have been reported to induce DNA fragmentation in mussels (*Mytilus galloprovincialis*) (Avio et al. 2015; Pittura et al. 2018; Revel et al. 2019), *Scrobicularia plana* (Ribeiro et al. 2017), and *Neocaridina davidi* (Berber 2019), and the intestinal cell line (Bussolaro et al. 2019).

The electrophoretic techniques (e.g., SDS-PAGE) are promising tools for identifying protein profile in response to stress and sublethal level of different pollutants (Muhammad et al. 2018; Paruruckumani et al. 2015a).

The Nile Tilapia (*Oreochromis niloticus*) is an African freshwater cichlid and one of the world’s most important food fishes (Soliman 2017). So, the present study aimed to examine the effects of microplastics on oxidative stress response, DNA fragmentation, and proteinogram of the popular fish (*Oreochromis niloticus*) early juvenile.

## Materials and methods

### Microplastics

Microplastics (with > 90% of microplastics > 100 nm in size) purchased from Toxemerge Pty Ltd., Australia).

### Fish exposure

Tilapia (*Oreochromis niloticus*) early juvenile (weight 4.35 ± 0.067, length 3.28 ± 0.12) were obtained from Aquaponics unit (Al Azhar University) and transported to the Fish Biology Laboratory. The physicochemical properties of test water (conductivity 260.8 μ M cm^−1^, pH 7.4, dissolved oxygen 6.9 mg L^−1^, temperature 28.5 °C, photoperiod 12:12 light:dark). Four groups of fishes (30 fish per each) were used: one control, three MPs-exposed groups as 1 mg/L of MPs, 10 mg/L of MPs, and 100 mg/L of MPs respectively for 15 days and 15 days of recovery according to Katzenberger and Thorpe (2015). At the end of the experiment, six fish from each group were randomly chosen and benumbed using ice to lessen the stress owing to processing. Blood was collected from the caudal vein for antioxidant enzymes and lipid peroxidation measurements as well as liver tissue for DNA fragmentation assay and muscle tissue for SDS-PAGE.

### Measurement of antioxidants biomarkers

Antioxidants kits were purchased from Biodiagnostic Company, Cairo, Egypt. The MPs stock solution was prepared according to (Hamed et al. 2019). Superoxide dismutase (SOD) was measured based on its ability to inhibit the phenazine methosulphate-mediated reduction of nitroblue tetrazolium dye to form a red product (Nishikimi et al. 1972). Catalase (CAT) was determined based on the fact that 3,5-dichloro-2-hydroxybenzene sulfonic acid could rapidly terminate the degradation reaction of hydrogen peroxide catalyzed by CAT and react with the residual hydrogen peroxide to generate a yellow product (Aebi 1984). Total antioxidant capacity (TAC) was measured according to protocol given by (Koracevic et al. 2001). Total peroxide (TPX) was assessed following the procedure of (Harma et al. 2005) and calculated from the standard curve constructed using standard concentrations.

### Plasma oxidative stress index, lipid peroxidation, and DNA fragmentation

It is the percentage ratio of TPX content to TAC concentration, and measured according to the following equation (Harma et al. 2005): OSI = (TPX, μM/L)/(TAC, μM/L) × 100.

Malondialdehyde (MDA) was measured according to the thiobarbituric acid reaction (Ohkawa et al. 1979). Three hundred milligrams of liver tissue was stored at 80 °C for measurement of DNA fragmentation according to Mekkawy et al. (2010). Tissues were homogenized in cold phosphate buffer saline (0.1 M; pH 7.4) using a Pottere Elvejhem glass/Teflon homogenizer. Thereafter, homogenates filtration and centrifugation were performed (10 min at 4 °C; 1600 rpm). DNA fragmentation was determined based on (Kurita-Ochiai et al. 1999).

### SDS-PAGE technique

Portions of muscles (~ 0.1 g fresh weight) of each fish were suspended in 1.0 ml lysing buffer, heated at 100 °C for 5 min, centrifuged at 10,000 rpm for 30 min, and 50 μL of extracted protein in each treatment was used for protein analysis using SDS-PAGE according to (Laemmli 1970) in the first dimension. The low molecular weight standards (BioBasic, USA) were run concurrently. The protein molecular mass and densitometric analysis of protein bands were determined using Gel-Pro Analyzer package V3.1 for Windows XP/NT (Media Cybernetica 1993–97).

### Statistical analysis

All data were analyzed using the SPSS package (SPSS 1998) at the 0.05 significance level. Data were tested for normality (Shapiro-Wilk test) and our data were normal. Then, data were tested for homogeneity of variances (Levene’s test) using one-way analysis of variance (ANOVA). In the case of variance equality, Fisher’s LSD post hoc test was used to compare treated groups against control. In cases of variance inequality, Dunnett’s post hoc test was used to compare treated groups against control.

### Ethical statement

Experimental setup and fish handling were approved by the Research, Ethical Committee of the Faculty of Science, Assuit University, Assuit, Egypt.

## Results

### Antioxidants biomarkers

The activities of superoxide dismutase (SOD), catalase (CAT), total peroxides (TPX), malondialdehyde (MDA), and oxidative stress index (OSI) showed significant increase (*P* < 0.05) after exposure to 1, 10, and 100 mg/L of microplastics for 15 days compared to the control group (Table 1). On the other hand, the activity of total antioxidant capacity (TAC) displayed a significant decrease (*P* < 0.05) after exposure to 1, 10 and 100 mg/L of microplastics for 15 days (Table 1). After the recovery period, the activities of superoxide dismutase (SOD), catalase (CAT), total peroxides (TPX), malondialdehyde (MDA), and total antioxidant capacity (TAC) returned to normal levels in 1 mg/L of MPs-exposed group. While oxidative stress index (OSI) did not recover to normal levels in all MPs-exposed groups (Table 1).

**Table 1:** Effect of microplastics (MPs) exposure and recovery for 15 days on antioxidants enzymes of the tilapia (*Oreochromis niloticus*) early juvenile

Biomarkers	Groups							
	Exposure period				Recovery period			
	Control	1 mg/L of MPs	10 mg/L of MPs	100 mg/L of MPs	Control	1 mg/L of MPs	10 mg/L of MPs	100 mg/L of MPs
SOD (IU/L)	11.01 ± 0.11^a^	11.62 ± 0.07^b^	11.88 ± 0.04^b^	11.95 ± 0.02^b^	10.35 ± 0.03^a^	11.50 ± 0.07^ab^	11.74 ± 0.03^bc^	11.87 ± 0.01^c^
CAT (IU/L)	10.23 ± 0.11^a^	10.72 ± 0.06^ab^	10.83 ± 0.04^bc^	11.03 ± 0.04^c^	10.23 ± 0.08^a^	10.52 ± 0.05^ab^	10.74 ± 0.05^bc^	10.82 ± 0.05^c^
TPX (μM/L)	1.49 ± 0.02^a^	1.58 ± 0.01^ab^	1.65 ± 0.01^bc^	1.83 ± 0.05^c^	1.44 ± 0.04^a^	1.54 ± 0.02^a^	1.59 ± 0.02^ab^	1.69 ± 0.03^b^
OSI (%)	147.21 ± 2.86^a^	163.72 ± 2.5^b^	179.87 ± 2.46^c^	207.50 ± 6.11^d^	142.88 ± 4.16^a^	153.04 ± 1.81^b^	163.4 ± 1.85^c^	188.33 ± 2.32^d^
TAC (μM/L)	1.01 ± 0.01^a^	0.97 ± 0.01^ab^	0.92 ± 0.01^bc^	0.88 ± 0.00^c^	1.01 ± 0.00^a^	1 ± 0.00^a^	0.97 ± 0.01^a^	0.90 ± 0.01^b^

Data are represented as mean ± SE. Values with different superscript letter in the same row for each parameter are significantly different (*P* < 0.05)

### Lipid peroxidation and DNA fragmentation

**Fig. 1:**
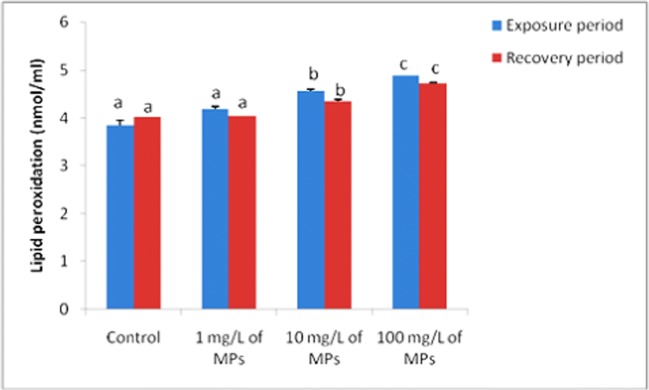
Lipid peroxidation (nmol/ml) in tilapia (*Oreochromis niloticus*) early juvenile after microplastics (MPs) exposure and recovery for 15 days

**Fig. 2:**
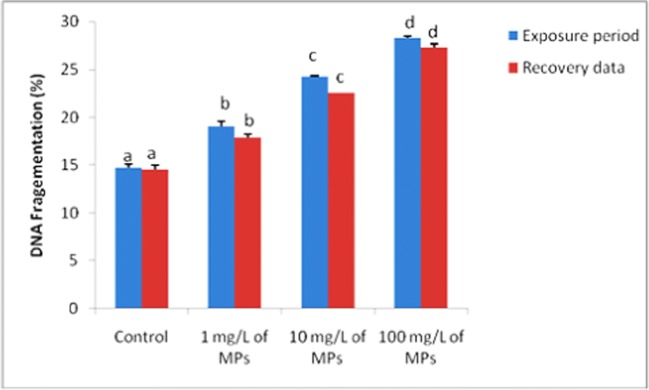
Liver DNA fragmentation (%) in tilapia (*Oreochromis niloticus*) early juvenile after microplastics (MPs) exposure and recovery for 15 days

Lipid peroxidation and DNA fragmentation showed significant increase (*P* < 0.05) after exposure to 1, 10, and 100 mg/L of microplastics for 15 days compared to the control group (Figs. 1 and 2). After the recovery period, lipid peroxidation and DNA fragmentation returned to normal levels in 1 mg/L of MPs-exposed group (Figs. 1 and 2).

### The electrophoretic pattern of muscle proteins

**Fig. 3:**
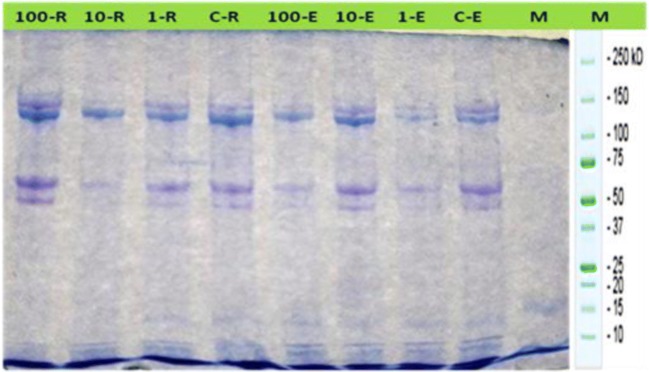
Electropherogram of muscle proteins of the tilapia (*Oreochromis niloticus*) early juvenile after microplastics (MPs) exposure and recovery for 15 days

The electrophoretic pattern showed that the total number of muscle protein bands in control group was 8 bands (Table 2 and Fig. 3). The number of protein bands decreased to 7 and 5 in muscle after exposure to 1, 10, and 100 mg/L of microplastics for 15 days, respectively (Table 2 and Fig. 3). After the recovery period, the total number of muscle protein bands returned to normal levels in 1 mg/L of MPs-exposed group (Table 2 and Fig. 3).

**Table 2:** Molecular weight (in kda) of muscle proteins of the tilapia (*Oreochromis niloticus*) early juvenile after microplastics (MPs) exposure and recovery for 15 days

Rows	Lanes								
	Marker	Exposure period				Recovery period			
		Control	1 mg/L of MPs	10 mg/L of MPs	100 mg/L of MPs	Control	1 mg/L of MPs	10 mg/L of MPs	100 mg/L of MPs
r1	233					229			
r2					217			212	
r3			207	209	208		206		206
r4	205	204		202		205		202	
r5		196	197		200	196	198	196	199
r6	186								
r7	164						163		
r8		146	147	149	150	145	147	145	146
r9	136	135	135		135	133	132	130	
r10	115								
r11	85								
r12	67								
r13	52	49	52			49			
r14	34	35	35	34		36	32	30	33
r15		24	29	25	28	30	26	24	23

## Discussion

Antioxidant biomarkers were used to detect various environmental stressors effects in some aquatic organisms (Hook et al. 2014). In the present study, the activities of superoxide dismutase (SOD), catalase (CAT), total peroxides (TPX), malondialdehyde (MDA), DNA fragmentation, and oxidative stress index (OSI) showed a significant increase while the activity of total antioxidant capacity (TAC) showed a significant decrease after exposure to 1, 10, and 100 mg/L of microplastics for 15 days compared to the control group. Microplastics that prompted disturbance in antioxidant enzymes have been reported in different aquatic organisms (Prinz and Korez 2020) including early juveniles of the common goby (*Pomatoschistus microps*) after the concurrent exposure to MP and Cr (VI) (Luis et al. 2015) and monogonont rotifer (*Brachionus koreanus*) exposed to 10 mg L^−1^ PS-MPs (Jeong et al. 2016). Moreover, the activity of catalase (CAT) was increased in zebrafish after exposure to 5-μm polystyrene microplastics (Lu et al. 2016). Similarly, catalase (CAT) activity was induced in sheepshead minnows (*Cyprinodon variegatus*) exposed to 50 mg/L irregular microplastics (Choi et al. 2018). Also, Paul-Pont et al. (2016) reported that the activity of SOD was elevated after co-exposure to MPs and fluoranthene in the marine mussels (*Mytilus* spp.) compared to the single groups (control; FLU and micro-PS). Moreover, activities of SOD increased in red tilapia exposed to 0.1 μm PS-MPs (Ding et al. 2018). Furthermore, Qu et al. (2019) observed that the activity of SOD was induced after co-exposure to micro-PS + venlafaxine in the loach (*Misgurnus anguillicaudatus*). The SOD activities induced in the liver of red tilapia (*Oreochromis niloticus*) after exposure to polystyrene microplastics (Zhang et al. 2018). In addition, CAT and SOD activities were elevated in the gut of the zebrafish exposed to MPs (Qiao et al. 2019).

Lipid peroxidation levels were induced in juveniles of the marine fish (*Pomatoschistus microps*) after exposure to PE-MPs (Ferreira et al. 2016). Also, the higher LPO levels were observed in the brain of *D. labrax* juveniles treated with microplastics (Barboza et al. 2018). Furthermore, higher LPO levels were observed in the freshwater bivalve (*Corbicula fluminea)* treated with microplastics (Oliveira et al. 2018). The oxidative stress and lipid peroxidation may be assigned to either malnutrition or food digestion inhibition due to large-sized MPs that were ingested by the fish (Lu et al. 2016) as well as additives that are commonly incorporated into plastics during manufacture to change their properties or extend the life of the plastic which include endocrine-disrupting chemicals such as polybrominated diphenyl ethers, nonylphenol, phthalates, and the constituent monomer bisphenol-A. If these chemicals leach out of the stored plastics, they could potentially affect the physiology of the animal (Katzenberger and Thorpe 2015) and this concept is supported by the first part of this study as hemotoxic effects of MPs (Hamed et al. 2019). Oxidative stress index (OSI) is a comparative indicator illustrating the interaction among the free-radical inducing agents and their antioxidants system (Sayed and Abu Khalil 2016). Oxidative stress index (OSI) induction has been reported in different models as arsenite toxicity in goldfish (Bagnyukova et al. 2007), methyltestosterone effects in Nile Tilapia (*Oreochromis niloticus*) (Sayed and Abu Khalil 2016).

In contrary to our results, the activity of SOD decreased after co-exposure to Cd and MPs in zebrafish (Lu et al. 2016). In addition, the activity of CAT declined in zebrafish treated with polystyrene microplastics (Wan et al. 2019). This decline in antioxidant enzymes was explained by energy expenditure of oxidative stress in response to microplastic exposition. Moreover, an elevation in TAC was detected in *Mytilus galloprovincialis* treated with 50 mg L^−1^ PS (Brandts et al. 2018; Pittura et al. 2018), while it was observed that virgin and contaminated low-density polyethylene did not induce TAC and lipid peroxidation in mussels.

Microplastics have been reported to induce DNA fragmentation in mussels *Mytilus galloprovincialis* (Avio et al. 2015; Pittura et al. 2018; Revel et al. 2019), *Scrobicularia* (Ribeiro et al. 2017), *Neocaridina davidi* (Berber 2019), and the intestinal cell line (Bussolaro et al. 2019). The genotoxicity of microplastics can be related to either the direct interaction with DNA or indirect mechanism by free radicals overproduction and oxidative stress (Manke et al. 2013). On the other hand, DNA strand break levels remain steady in (*Mytilus galloprovincialis)* treated with microplastics (Pittura et al. 2018). Also, virgin polystyrene microbeads do not produce genetic damage in freshwater zebra mussel (*Dreissena polymorpha*) (Magni et al. 2018).

In this study, the electrophoretic pattern showed that total number of muscle protein bands in control group was 8 bands. The number of protein bands decreased to 7 and 5 after exposure to 1, 10, and 100 mg/L of microplastics for 15 days, respectively. The change in the protein subunit band patterns may be due to change in the turn over (synthesis/degradation) of various proteins (Paruruckumani et al. 2015b). Also, Patterson (1976) reported that the pollutants inhibit protein synthesis through interaction with nucleic acids where our results were matched with this concept. Similar reduction in the number of banding pattern was observed in the muscle and gills of *Lates calcarifer* after exposure to copper (Paruruckumani et al. 2015b). Some pollutants (e.g., CuSo_4_, malathion, and paraquat) prompted disappearance of specific blood protein fractions in *Oreochromis niloticus* (Sharaf-Eldeen and Abdel-Hamid 2002). Tripathi and Shukla (1990) observed alterations in the cytoplasmic protein pattern of fish *Clarias batrachus* by performing electrophoresis of the cytoplasmic protein fractions of the liver and the skeletal muscle exposed to endosulfan and methyl parathion for 28 days. Badaway and El-Serafy (1998) reported that *Clarias gariepinus* collected from different polluted water bodies showed disappearance of specific serum protein fractions and others were polymorphic.

In the present study, after the recovery period, the activities of superoxide dismutase (SOD), catalase (CAT), total peroxides (TPX), total antioxidant capacity (TAC), malondialdehyde (MDA), DNA fragmentation, and the electrophoretic pattern of muscle proteins returned to normal levels in 1 mg/L of MPs-exposed group, while oxidative stress index (OSI) did not recover to normal levels in all MPs-exposed groups. This may be explained by either the inability of animal tissues to eliminate microplastics or the capability to regain after recovery period (Ribeiro et al. 2017). Moreover, Paul-Pont et al. (2016) observed that after the elimination period, some antioxidant enzymes still significantly highly reflected the need for a greater capability to neutralize free radicals.

## Conclusion

Microplastics exposure caused alterations in the antioxidants biomarkers (SOD, CAT, and TAC), DNA damage, and alteration in the muscle protein profile of tilapia early juvenile which may be regarded as a reason for fish mortality. The MPs have a dose-dependent effect.
